# Supplementary material for: Dose- and Time-Dependent Effects of Radiofrequency Electromagnetic Field on Adipose Tissue: Implications of Thermoregulation and Mitochondrial Signaling
Source: Int J Mol Sci. 2023 Jun 25;24(13):10628. doi: 10.3390/ijms241310628 (PMC10342026; doi:10.3390/ijms241310628)
Supplement: Supplementary file 1 [file ijms-24-10628-s001.zip › ijms-2462945-supplementary.pdf]

# Supplementary material S1 – Housekeeping genes formalisation

## WAT

| Method | 1 | 2 |
|---|---|---|
| Delta CT | gapdh | 18S |
| BestKeeper | 18S | gapdh |
| Normfinder | gapdh | 18S |
| Genorm | 18S \| gapdh | |
| **Recommended comprehensive ranking** | **gapdh** | **18S** |

## BAT

| Ranking Order (Better--Good--Average) | | |
|---|---|---|
| Method | 1 | 2 |
| Delta CT | 18S | gapdh |
| BestKeeper | 18S | gapdh |
| Normfinder | 18S | gapdh |
| Genorm | gapdh \| 18S | |
| **Recommended comprehensive ranking** | **18S** | **gapdh** |

**Table S1 Stability of housekeeping genes using the programs BestKeeper, Normfinder, and Genorm in WAT and BAT.**
